# Anti-Osteoclastogenesis Potential of Cocoa Pod Husk (*Theobroma cacao L*.) Extract: In Silico and *In Vivo* Study

**DOI:** 10.1590/0103-6440202406015

**Published:** 2024-12-02

**Authors:** Yani Corvianindya Rahayu, Ernie Maduratna Setiawatie, Retno Pudji Rahayu, S Siswandono, Retno Indrawati, Hendrik Setia Budi, Hari Basuki Notobroto, Rahmah A. Alwasilah Darojah

**Affiliations:** 1 Doctoral Study Program in Dental Science, Faculty of Dental Medicine, Universitas Airlangga, Surabaya, Indonesia; 2 Department of Oral Biology, Faculty of Dentistry, Universitas Jember, Indonesia; 3 Department of Periodontology, Faculty of Dental Medicine, Universitas Airlangga, Surabaya, Indonesia; 4 Department of Oral Pathology and Maxillofacial, Faculty of Dental Medicine, Universitas Airlangga, Surabaya, Indonesia; 5 Department of Pharmacology, Faculty of Pharmacy, Universitas Airlangga, Surabaya, Indonesia; 6 Department of Oral Biology, Faculty of Dental Medicine, Universitas Airlangga, Surabaya, Indonesia; 7 Department of Epidemiology, Biostatistics and Population Studies, and Health Promotion, Faculty of Public Health, Universitas Airlangga, Surabaya, Indonesia; 8 Faculty of Dentistry, Universitas Jember, Indonesia

**Keywords:** in silico, osteoclastogenesis, RANKL, TNF-α, Theobroma cacao

## Abstract

Periodontitis is a common chronic inflammatory disease characterized by alveolar bone loss. The high polyphenol content in cocoa pod husk (*Theobroma cacao* L) has the potential to influence bone metabolism and contribute to the inhibition of bone resorption. The aim of this study was to analyze the anti-osteoclastogenesis potential of cocoa pod husk (*Theobroma cacao* L.) in both *in silico* and *in vivo* study. An analysis of the anti-osteoclastogenesis potential of *T. cacao* bioactive compounds was conducted using molecular docking simulations. Thirty male Wistar rats (*Rattus novergicus*) were randomly assigned to control negative groups (placebo gel), control positive groups (2% doxycycline gel), and treatment groups (10% cocoa pod husk (CPH) extract gel), with measurements taken on days 7 and 14. Wistar rats were induced with 0.05 ml of *P. gingivalis* at a concentration of 2x10^9^ CFU/ml intrasulcularly in the maxillary molar to achieved in periodontitis. The number of osteoclasts was observed by hematoxylin and eosin staining, the level of TNF-α was assessed by enzyme-linked immunosorbent assay, and the expression of RANKL was evaluated by immunohistochemical staining. Data were analyzed using One-way ANOVA to examine the differences between the groups. The in silico study showed that the catechin, epicatechin, quercetin, and procyanidin B2 had a strong binding affinity for TNF-α and RANKL. Administration of 10% CPH reduced the number of osteoclasts (p<0.05), TNF-α level on days 7 and 14 (p<0.05), and RANKL expression on day 7 (p<0.05) in experimental rats with periodontitis. Administering 10% CPH inhibited osteoclastogenesis in the experimental periodontitis rats.

## Resumo

Periodontite é uma doença inflamatória crônica comum caracterizada pela perda de osso alveolar. O alto teor de polifenóis na casca do cacau (*Theobroma cacao* L) tem o potencial de influenciar o metabolismo ósseo e contribuir para a inibição da reabsorção óssea. O objetivo deste estudo foi analisar o potencial anti-osteoclastogênico da casca do cacau (*Theobroma cacao* L.) em um estudo *in silico* e *in vivo*. Uma análise do potencial anti-osteoclastogênico dos compostos bioativos do *T. cacao* foi realizada por simulações de docking molecular. Trinta ratos Wistar machos (*Rattus novergicus*) foram aleatoriamente selecionados em grupos controle negativo (gel placebo), controle positivo (gel de doxiciclina a 2%) e grupos de tratamento (gel com extrato de casca de cacau a 10%), e as medições foram feitas nos dias 7 e 14. Ratos Wistar foram induzidos com 0,05 ml de *P. gingivalis* a uma concentração de 2x10^9 UFC/ml de forma intrasulcular no molar maxilar para atingir a periodontite. O número de osteoclastos foi observado por coloração de hematoxilina e eosina, o nível de TNF-α foi observado por ensaio imunoenzimático, e a expressão de RANKL foi observada por coloração imunohistoquímica. Os dados foram analisados usando ANOVA de uma via para examinar a diferença entre os grupos. O estudo *in silico* mostrou que a catequina, epicatequina, quercetina e procyanidina B2 tinham uma forte afinidade de ligação para TNF-α e RANKL. A administração de 10% de CPH reduziu o número de osteoclastos (p<0,05), o nível de TNF-α nos dias 7 e 14 (p<0,05) e a expressão de RANKL no dia 7 (p<0,05) em ratos experimentais com periodontite. A administração de 10% de CPH inibiu a osteoclastogênese nos ratos com periodontite experimental

## Introduction

Periodontitis is a common chronic inflammatory disease that affects 50% of the adult population worldwide. It is characterized by alveolar bone loss, leading to gingival recession and periodontal pocket formation ^1^ ^,^ ^2^. Patients with periodontitis experience a declining quality of life, particularly concerning oral health. Oral problems can have serious systemic effects by disseminating pathogenic bacteria into the blood and bones and causing a pro-inflammatory state that may lead to systemic diseases ^3^ ^,^ ^4^. Red complex bacteria, such as *Porphyromonas gingivalis*, play a key role in the polymicrobial synergy and dysbiosis of the subgingival microbiome. The virulence factors of *P. gingivalis* stimulate the host’s immune cells to release inflammatory mediators, impacting on apoptosis, tissue damage, and inflammatory reactions which results in periodontal pockets, bleeding on probing, and bone destruction. Further consequences include increased osteoclast activity and decreased osteoblast activity in the alveolar bone ^5^ ^,^ ^6^.

Interleukin-1β and tumor necrosis factor-α (TNF-α) serve as specific mediators of inflammation in periodontal disease, regulating the expression of receptor activator of nuclear factor kappa B ligand (RANKL) and osteoprotegerin (OPG) in osteoblasts ^7^. Patients with chronic periodontitis exhibit higher RANKL levels in periodontal tissues compared to healthy individuals. RANKL inhibition by osteoprotegerin (OPG), a RANKL inhibitor, blocks alveolar bone loss in rats with periodontitis. Chronic periodontitis patients also show elevated TNF-α levels in gingival serum. TNF-α is a major inflammatory cytokine that plays a significant role in periodontal destruction and is highly toxic to the host ^8^.

TNF-α initiates various biological signaling pathways by binding to two specific receptors, tumor necrosis factor receptor type I (TNFR1) and tumor necrosis factor receptor type II (TNFR2). In a normal physiological environment, TNFR2 is expressed primarily in immune cells, endothelial cells, and neural cells, whereas TNFR1 is present in almost all nucleated cells and is crucial for triggering immune signaling pathways. Upon receptor binding, TNF-α activates several important signaling pathways, including c-Jun N-terminal kinase, mitogen-activated protein kinase (MAPK), extracellular signal-regulated kinase (ERK), and the transcription factor NFκB ^9^.

Osteoclastogenesis is mediated by macrophage colony-stimulating factor (M-CSF), which promotes osteoclast precursor proliferation, and by RANKL, which drives osteoclast differentiation ^10^. RANKL’s binding to RANK will induce differentiation of osteoclast precursor cells into mature osteoclasts. Osteoclastogenesis activity for absorbing dead bone cells and replacing them with new bone cells is increased. A decrease in inflammatory cells indicates the onset of osteoblastogenesis, a process characterized by the binding of RANK and OPG, which increases osteoblast activity in the alveolar bone ^6^ ^,^ ^11^.

In periodontitis, an increase in pro-inflammatory cytokines and ROS causes a homeostatic imbalance between osteoclastogenesis and osteoblastogenesis. This imbalance is indicated by increased RANK-RANKL binding and decreased RANK-OPG binding, leading to a decline in bone regeneration activity ^12^. The gold standard treatments for periodontitis include scaling and root planing. In the progressive stage, bone destruction is frequently observed. Therefore, administering antibiotics as part of the treatment can enhance the effectiveness of periodontal therapy ^13^. Systemic administration of antibiotics carries a higher risk of side effects compared to topical administration, which allows for direct binding of the drug to the receptors. Herbal medicines offer an alternative with various drug-like biological activities and generally lower side effects compared to chemical drugs ^14^ ^,^ ^15^.

Cocoa (*Theobroma cacao L.*) presents a potential natural treatment for periodontitis. It is a high-yield plantation crop in Indonesia. However, the increasing amount of cocoa pod waste from cocoa manufacturing can pose environmental concerns if not managed properly. Research has shown that a 10% cocoa pod husk extract has low cytotoxicity and the potential to reduce levels of COX-2 and MMP-8 in rats with periodontitis ^18^ ^,^ ^19^. Several phytochemical studies have demonstrated that cocoa pod husk contains secondary metabolites such as alkaloids, saponins, and polyphenols, including flavonoids and tannins. Polar extract analysis of cocoa pod husk using LC-MS has identified several polyphenols, such as catechin, epicatechin, quercetin, and procyanidin B2 ^16^ ^,^ ^17^. Polyphenols, particularly flavonoids, play a crucial role in anti-inflammatory and antioxidant properties. Flavonoids also promote osteoblast differentiation and reduce the number of osteoclasts ^20^.

Given the limited research on the bioactive compounds in cocoa pod husk (*T. cacao*) and their potential for periodontal tissue regeneration, especially in inhibiting alveolar bone resorption, an in silico and in vivo study was conducted for further exploration. The study utilized computer simulation methods to design, predict, , and uncover the potential of the drug. Molecular docking employed in the *in silico* study to align ligands with receptors, considering the properties of both ^21^. This study aimed to analyze the anti-inflammatory role of cocoa pod husk (*Theobroma cacao L.*) in inhibiting osteoclastogenesis in rats with periodontitis and to predict the interactions between the compounds in cocoa pod husk and the target proteins TNFR and RANKL. The in vivo study used rat models with periodontitis to observe the TNF-α level in gingival crevicular fluid, the number of osteoclasts, and the expression of RANKL following the application of a 10% cocoa pod husk (*Theobroma cacao L.*) extract gel.

## Materials And Methods

### Extraction of cocoa pod husk (Theobroma cacao L.)

The cocoa pod husk used was *Theobroma cacao* L. of the forastero type, identified at the Plant Laboratory of Politeknik Negeri Jember. The fruit was characterized by its even yellow skin. All parts of the cocoa pod were used in the extraction process. After cleaning and air-drying at room temperature, the cocoa pod husk was further dried in an oven at 50°C for 48 hours and then ground into a powder using a blender. The extraction process was performed using an ultrasonic bath (Elma S100h, Germany) with 70% ethanol as the solvent, in three sequential extractions with ratios of cocoa pod husk powder to solvent of 1:4, 1:2, and 1:1.5, each carried out for 30 minutes. To concentrate the extract, a rotary evaporator (B-one RE200VN, USA) was used, followed by further evaporation using an oven at 50^o^C. The final extract concentration of 100 mg/mL (10%) was obtained as a single dose, following previous findings that 10% cocoa pod husk ethanol extract has a low level of cytotoxicity and a quite high antioxidant content ^17^. 

### Cocoa pod husk extract gel preparation

The preparation of placebo gel was carried out by pouring 48 mL of aquadest into a mortar and adding two grams of sodium carboxymethyl cellulose (CMC-Na), left for around 10-15 minutes. Both ingredients were stirred to reach homogeneity until it turned into CMC-Na placebo gel (4%). The preparation of 2% doxycycline gel used as a positive control involved mixing 0.5 grams of doxycycline powder and 24.5 grams of placebo gel and stirring them until well-mixed. For the 10% ethanol extract gel from cocoa pod husk, 2.5 grams of 100 mg/mL cocoa pod husk ethanol extract was mixed with 22.5 grams of placebo gel and stirred until thoroughly mixed ^22^.

### Preparation of P. gingivalis suspension

One inoculation loop of *P. gingivalis* ATCC 33277 was cultured in TSB medium and incubated for 2 x 24 hours at 37^o^C. The suspension was made by taking one inoculum loop of *P. gingivalis* from the culture preparation and dissolving it in 1 cc of saline/PZ solution in a tube. The mixture was homogenized by centrifugation and measured at a concentration of 2 x 10^9^ CFU/mL, equivalent to the 0.5 McFarland standard, using a spectrophotometer (Shidmadzu UV-2600i, Japan). 

### Ethical approval and experimental animals 

Thirty male Wistar rats, 20 weeks old, weighing 200-220 g, were housed in a room under controlled temperature with a 12-hour light-dark cycle and a humidity of 55 to 70%. All rats were kept for one week for adjustable feeding before the experiment, and food and water were provided *ad libitum*. The subjects were divided into three groups: a negative control group (CMC-Na gel); a positive control group (doxycycline gel); and a treatment group (ethanol extract gel of cocoa pod husk at 100 mg/mL). Observations were made on days 7 and 14 (n = 5 per group). This study was approved by the Ethics Committee of Airlangga University (No. 1406/HRECC/FDOM/XII/2023) and was conducted according to the ARRIVE guidelines for the care and use of animals in experimental procedures ^23^.

 Anesthetizing was performed using a cocktail of 0.2 mL of ketamine + xylazine, administered intramuscularly while each rat was placed in a dental chair designed for rodents. Induction of 0.05 mL of *P. gingivalis* at a concentration of 2 x 10^9^ CFU/mL was conducted intrasulcularly in the buccal lobe of the maxillary first molar using a 30-gauge tuberculine syringe. *P. gingivalis* induction was performed three times a week alternately for two weeks to achieve periodontitis in rats. The placebo CMC-Na gel, 2% doxycycline gel, and 10% cocoa pod husk extract gel were applied topically once every morning for seven days. Prior to each gel application, the experimental animals were anesthetized with the ketamine-xylazine cocktail. The gel was applied topically at a volume of 0.05 mL using a syringe with a gastric tube ^24^.

### In silico molecular docking analysis of TNFR and RANKL

Four active compounds of *Theobroma cacao* Linn: catechin, epicatechin, quercetin, and procyanidin B2 were selected for molecular docking simulation with tumor necrosis factor receptor (TNFR) and receptor activator of nuclear factor-kβ ligand (RANKL). The 3D structures of the selected molecules were retrieved from PubChem (https://pubchem.ncbi.nlm.nih.gov/). The downloaded molecule structures were converted into the pdb format using Pymol (Schr dinger Inc., LLC), and their energies were minimized using Open Babel in PyRx 0.8 (The Scripps Research Institute, California). Energy minimization is often performed before molecular docking to achieve a stable conformation that closely resembles the biological system's original state. The 3D structures of TNFR (6BWV) and RANKL (3URF) were obtained from the Protein Data Bank (PDB) (https://www.rcsb.org/) ^21^. Water molecules were removed from the protein structures following standard procedures in Pymol. Discovery Studio v20 was used to evaluate the amino acids in TNFR and RANKL that interacted with catechin, epicatechin, quercetin, and procyanins B2. The three-dimensional (3D) visualization of molecular docking results and protein-ligand interactions was performed using PyMOL ^25^.

### Enzyme-linked immunosorbent assay of TNF-α

Measurements of TNF-α protein levels in gingival crevicular fluid (GCF) samples were taken using paper points number 15 for 30 seconds. The paper point was positioned horizontally in the gingival groove area of the distobuccal part of the maxillary first molar. GCF samples must be taken carefully so as not to cause any injury to the gingival groove area, which in turn would cause contamination. The paper points were then placed into 0.5 mL Eppendorf tubes and stored at -20°C until the enzyme-linked immunosorbent assay was performed. Each Eppendorf tube supplemented with 50 µL of 0.02 M phosphate buffer solution (pH 7.0-7.2), followed by 2000-3000 rpm centrifugation at room temperature (18-25^o^C) for 20 minutes. The resultant protein levels of TNF-α (ABTS, PeproTech^®^) were read using an ELISA reader (Multiskan FC, Thermo Fisher, USA ) in the 450 nm wavelength for a maximum of 30 minutes after the addition of the stop solution ^26^.

### Observation of the number of osteoclasts

**Figure. 1:**
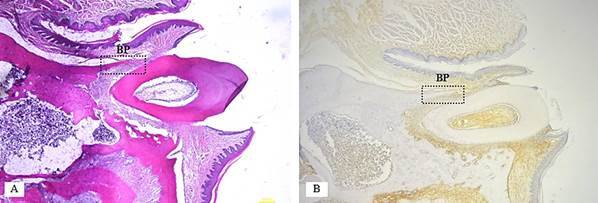
Histological features of alveolar bone resorption in the maxillary first molar of a Wistar Rat periodontitis model and measurement area in the buccal periosteum (BP). A) hematoxyllin eosin staining B) immunohistochemistry staining

Experimental animals were sacrificed by administering a lethal dose of anesthetic, consisting of 100 mg/kg ketamine and 2 mg/kg xylazine (2% Alfazyne, Alfasan International, Woerden, Netherlands) i.m ^24^. Tissues from the left maxillary molar region were then sampled and immediately fixed in 10% neutral buffered formalin for 24 hours. The samples were then decalcified with 10% formic acid (Sigma-Aldrich; Castle Hill, Australia) for 28 days. The tissue samples was processed by the paraffin embedding method, sectioned sagittally at a thickness of 5 μm using a microtome (Sakura Finetek, USA), and stained with hematoxylin and eosin (HE09-40R, TissuePro, USA). Observations were made under a binocular microscope (Olympus CX23, China) at 400x magnification. Three histological sections or fields were examined to measure the number of osteoclasts on the buccal side of the alveolar bone periosteum (Figure 1A). Measurements were confirmed using ImageJ software.

### Observation of the number of osteoclasts

Experimental animals were sacrificed by injection of a lethal dose of anesthetic using a mixtu

re of 100 mg/kg ketamine and 2 mg/kg xylazine (2% Alfazyne, Alfasan Int., Woerden, Netherlands) i.m ^24^. Tissues on the left maxillary molar were sampled and immediately fixed with 10% neutral formalin buffer for 24 hours. The sample was then decalcified with 10% formic acid (Sigma-Aldrich; Castle Hill, Australia) for 28 days. The tissue sample was processed by the paraffin embedding method, with sagittal incisions with a thickness of 5 μm using a microtome (Sakura Finetek, USA) and staining with hematoxylin and eosin stains (HE09-40R, TissuePro, USA). Observations were made under a binocular microscope (Olympus CX23, China) with 400x magnification. There were three histological sections or fields used to measure the number of osteoclasts on the buccal side of alveolar bone periosteum (Figure 1A). Confirmation was made with the Image J software.

### Immunohistochemical observation of the expression of RANKL 

Immunohistochemical staining was performed to observe the RANKL primary antibody (sc-7628, Santa Cruz Biotechnology). The number of osteoblasts expressing RANKL in the defective alveolar bone was assessed at 400x magnification using a binocular microscope. Three histological sections or fields were examined to measure the number of osteoblasts expressing RANKL on the buccal side of the alveolar bone periosteum (Figure 1B). The images were captured using Optilab Viewer 3.0 software. Osteoblasts were identified as cuboidal or columnar cells with adjacent positioning, round nuclei, and basophilic cytoplasm, and were analyzed using ImageJ software.

### Statistical analysis

The data were tabulated in Microsoft Excel, and the statistical analysis was performed with SPSS Software version 25.0 (IBM, Chicago, USA). The normality of the data was assessed using the Shapiro-Wilk test, and homogeneity of variances was evaluated with Levene's test. The dependent variables, considering time and treatment, were analyzed separately. A one-way analysis of variance (ANOVA) was used to examine differences between the groups. Statistical significance was determined if the p-value was less than 0.05 (p < 0.05).

## Results

Molecular docking simulation of TNFR and RANKL

A docking score of binding affinity with non-bonding interactions was used as the parameter for the binding interactions and affinity of the compounds, along with those of standard drugs. The docking scores with TNFR of catechin and quercetin showed the highest binding affinities (-6.5 kcal/mol), even greater than the standard drug, diclofenac. Molecular docking simulation results indicated that procyanidin B2 occupied the same binding site as diclofenac with the most amino acids (Ala222, Leu102, Leu147, and Ser145) found in ligand interactions with TNFR. It showed that it had an important role in the protein target, and the hydrogen bonds represented more stable and strong bonds between protein and ligand (Table 1).

**Table 1:** Molecular docking simulation and interaction of selected phytochemicals of *Theobroma cacao* with TNFR

Protein target	Compounds (ligands)	Binding affinity [kcal/mol]	Hydrogen bonds	van der Waals interaction
TNFR	Diclofenac (3033)	-6.2	G1n149, Val146	Ala222, Leu102, Leu147,Ser145, Val148
	Catechin (9064)	-6.5	Leu102, Trp226	Gln149, Gln22, Gly70, His224, Ile103, Leu147, Val152, Val71
	(-)-Epicatechin (72276)	-6.1	Leu102	Ala222, Ala225, Gln149, His224, Leu101, Ser145
	Quercetin (5280343)	-6.5	His224, Leu147	Gln227, Gly70, Ile103, Leu102, Ser145, Trp226, Val148, Val152, Val71
	Procyanidin B2 (122738)	-5.4	Arg223, His224, Trp226	Ala222, Gln149, Leu102, Leu147, Ser135, Val152, Val71

The docking scores with RANKL of epicatechin (-7.0 kcal/mol) and procyanidin B2 (-6.9 kcal/mol) exhibited the highest binding affinities, although these were lower than that of the standard drug, raloxifene. Molecular docking simulation results revealed that procyanidin B2 was located at the same binding site as raloxifene with the most amino acids (Ile249, Leu255, Leu60, Lys244, and Ser63) found in ligand interactions with RANKL (Table 2). 

**Table 2:** Molecular docking simulation and interaction of selected phytochemicalsn of *Theobroma cacao* with RANKL

Protein target	Compounds (ligands)	Binding affinity [kcal/mol]	Hydrogen bonds	van der Waals interaction
RANKL	Raloxifene (5035)	-7.6	Glu287, Tyr61	Arg284, Ile249, Leu255, Leu60, Met256, Pro64, Ser63, Tyr48
	Catechin (9064)	-6.7	Ser63, Thr254	Arg284, Glu58, Ile249, Leu255, Leu60, Lys248, Lys282, Ser246, Tyr61
	(-)-Epicatechin (72276)	-7.0	Cys59, Ser63, Thr254	Glu287, Glu58, Ile249, Leu255, Lys244, Lys248, Ser246, Tyr48, Tyr61
	Quercetin (5280343)	-6.8	Glu58, Tyr61	Arg284, Cys59, His253, Ile249, Leu255, Lys244, Phe281, Thr254
	Procyanidin B2 (122738)	-6.9	Tyr61	Ile249, Leu255, Leu60, Lys244, Lys257, Lys282, Met256, Pro64, Ser63m Thr254

**Figure 2:**
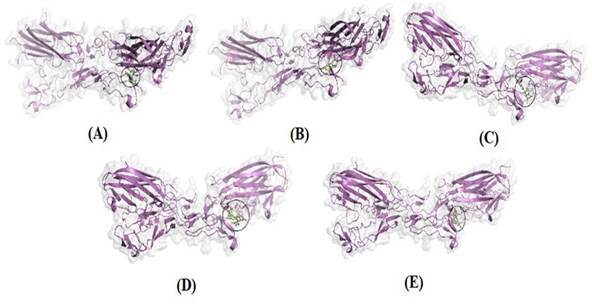
Three-dimensional (3D) visualization of docking results of four selected ligands with TNFR (A) Diclofenac, (B) Catechin, (C) (-)-Epicatechin (D) Procyanidin B2, (E) Quercetin.

**Figure. 3:**
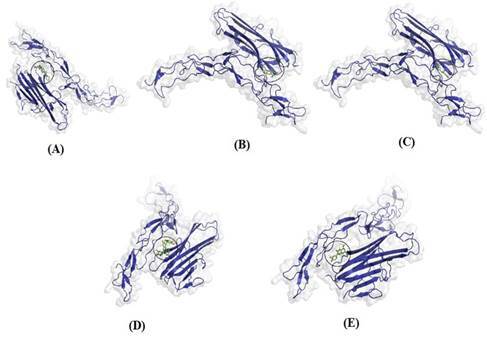
Three-dimensional (3D) visualization of docking results of four selected ligands with RANKL (A) Diclofenac, (B) Catechin, (C) (-)- Epicatechin (D) Procyanidin B2, (E) Quercetin.

All protein complexes that were docked are shown with 3D structures, transparent surfaces, and colored selections based on their constituent structures, and those docked for ligands were displayed with stick structures. The binding interactions between the docked molecules and the amino acid residues of the protein target TNFR are shown in Figure 2, and the binding interactions with the protein target RANKL are shown in Figure 3.

Based on the results of the molecular docking simulations of the protein targets and ligands, quercetin, catechin, (-)-epicatechin, and procyanidin, which are constituents of Theobroma cacao extract, exhibited similar binding modes and interactions to those of standard drugs. Therefore, these compounds are predicted to influence the activity of the target proteins, particularly in the inhibition of inflammation and osteogenesis. The identification of molecular interactions and binding sites on the docked protein-ligand complexes revealed that the bonding of all compounds with the target proteins involved non-covalent interactions, including van der Waals forces and hydrogen bonds.

### TNF-α level in gingival crevicular fluid

The protein levels of TNF-α are presented in Table 3. On day 7, the highest level of TNF-α (146.725 ± 5.91) was observed in the negative control group with placebo gel (NC). This was followed by the treatment group with cocoa pod husk extract gel (CPH) (99.441 ± 3.32) and the positive control group with 2% doxycycline gel (PC) (96.146 ± 5.39). These trends continued on day 14, with the highest TNF-α level still in the NC group (114.718 ± 4.72) and the lowest in the PC group (75.587 ± 4.86). The results indicated significant differences between the groups (p < 0.05).

**Table 3:** The level of TNF-α in gingival crevicular fluids of periodontitis rat

Day	TNF-α (mean ± SD)			p value
	NC	PC	CPH	
7	146.72 ± 5.91*	96.15 ± 5.39	99.44 ± 3.32	0.001
14	114.72 ± 4.72*	75.59 ± 4.86	79.52 ± 3.51	0.000

All data were collected in 3 groups (n=5). NC= Negative control group administering CMC-Na gel on day 7 and 14; PC= Positive control group administering doxycycline gel on day 7 and 14; CPH= Treatment group administering cocoa pod husk extract gel on day 7 and 14. *Significant p values (p ≤ 0.05).

### Number of osteoclasts in the alveolar bone of rats with periodontitis

**Figure. 4:**
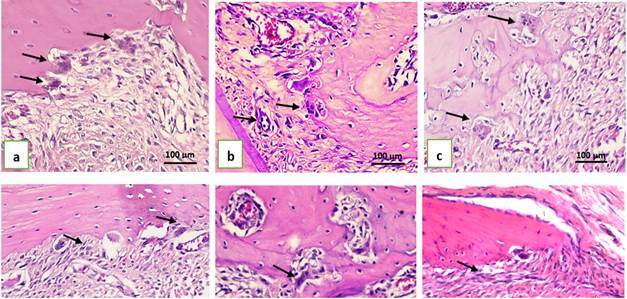
Histological features of alveolar bone’s periodontitis rats osteoclast in hematoxillin eosin staining at 400x magnification. a) negative control group (placebo CMC-Na gel) day 7; b) positive control group (doxycycline gel) day 7; c) treatment group (CPH 10% gel) day 7; d) negative control group (placebo CMC-Na gel) day 14; e) positive control group (doxycycline gel) day 14; f) treatment group (CPH 10% gel) day 14. Black arrows indicated osteoclast cells.

To evaluate the effect of CPH extract gel on osteoclast formation in rats with periodontitis, osteoclast cells were counted in three fields on the buccal side of the alveolar bone periosteum (Figures 3 and 4). On day 7 and day 14, the number of osteoclasts in the NC group was significantly higher than in the PC and CPH groups (p < 0.05). Significant differences were observed between the control and treatment groups on day 7 (p = 0.004) and day 14 (p = 0.047). However, post-hoc test results showed no significant differences between the PC and CPH groups on either day (Table 4).

**Table 4:** The number of osteoclast on alveolar bone of periodontitis rats

Day	Osteoclast (mean ± SD)			p value
	NC	PC	CPH	
7	6.25 ± 0.95*	3.50 ± 1.29*	3.00 ± 0.81*	0.004
14	3.50 ± 0.57*	1.75 ± 0.95	1.75 ± 0.95	0.047

All data were collected in 3 groups (n=5). NC= Negative control group administering CMC-Na gel on day 7 and 14; PC= Positive control group administering doxycycline gel on day 7 and 14; CPH= Treatment group administering cocoa pod husk extract gel on day 7 and 14. *Significant p values (p ≤ 0.05).

### Expression of RANKL in the alveolar bone of rats with periodontitis 

**Figure. 5:**
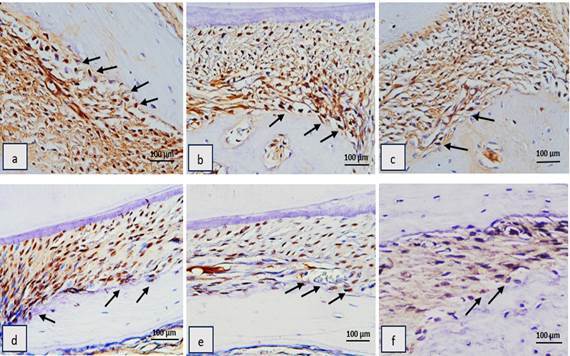
Histological features of RANKL immunohistochemistry at 400x magnification on alveolar bone of periodontitis rats. a) negative control group (placebo CMC-Na gel) day 7; b) positive control group (doxycycline gel) day 7; c) treatment group (CPH 10% gel) day 7; d) negative control group (placebo CMC-Na gel) day 14; e) positive control group (doxycycline gel) day 14; f) treatment group (CPH 10% gel) day 14. Black arrows indicated RANKL-expressing osteoblast cells.

The highest RANKL expression was observed in the NC group on day 7 (18.75 ± 2.62), while the lowest RANKL expression was found in the CPH group on day 14 (3.00 ± 0.81). The average RANKL expression in the negative control group on day 14 amounted to 15.5 ± 1.29, the average RANK expression in the positive control group on days 7 and 14 amounted to 12.00 ± 0.81 and 9.00 ± 0.81, respectively, and the average RANKL expression in the treatment group on day 7 amounted to 6 ± 0.81 (Figure 5). Based on the post-hoc LSD test results, there were significant differences (p < 0.05) in the number of osteoblasts expressing RANKL between the NC7, PC7, and CPH7 groups, as well as between the NC14, PC14, and CPH14 groups (Table 5).

**Table 5:** The expression of RANKL on alveolar bone of periodontitis rats

Day	RANKL (mean ± SD)			p value NC
	NC	PC	CPH	
7	18.75 ± 2.62*	12.00 ± 0.81*	7	18.75 ± 2.62*
14	15.50 ± 1.29*	9.00 ± 0.81*	14	15.50 ± 1.29*

All data were collected in 3 groups (n=5). NC= Negative control group administering CMC-Na gel on day 7 and 14; PC= Positive control group administering doxycycline gel on day 7 and 14; CPH= Treatment group administering cocoa pod husk extract gel on day 7 and 14. *Significant p values (p ≤ 0.05).

## Discussion

In this study, the selected compounds from cocoa pod husk demonstrated potential as inhibitors of TNFR and RANKL. Among all ligands, the strongest binding affinity for TNFR was shown by catechin and quercetin (-6.5 kcal/mol), followed by epicatechin (-6.1 kcal/mol). The affinity values ​​of catechin and quercetin for TNFR were even higher than that of the control compound, diclofenac. Meanwhile, the strongest binding affinity for RANKL was shown by epicatechin (-70 kcal/mol), which was close to the value of the control compound. Cocoa polyphenols are believed to influence bone metabolism by downregulating inflammatory mediators such as cytokines, which are associated with osteoclast differentiation and thus contribute to the inhibition of bone resorption. Several important molecular pathways involved in bone metabolism are targeted by polyphenols, such as the estrogen signaling (ER) pathway, the MAPK cascade, and the inflammatory pathways. Polyphenols have also been shown to inhibit osteoclastogenesis in bone loss pathologies ^27^ ^,^ ^28^. 

The highest level of TNF-α was observed in the NC7 group, with the PC7 and CPH7 groups showing lower levels, though the difference between these two groups was not statistically significant. This pattern persisted on day 14, where the level in the NC14 group was still high even though it had decreased compared to day 7. The levels in the PC14 group were lower than the those in the NC14 and CPH14 groups, but no significant statistical difference was found between PC14 and CPH14. It is suspected that the administration of CPH to rats with periodontitis provided a better anti-inflammatory effect than the administration of PC. Doxycycline, in addition to antibacterial properties, has also been widely reported to exhibit anti-inflammatory actions and show clinical benefits in the treatment of acute and chronic inflammatory disorders, including gingivitis and periodontitis ^29^. Doxycycline is able to downregulate the main inflammatory cytokines (IL-1, IL-6, TNF-α, and PGE2), stimulates fibroblast collagen production, and reduces osteoclast activity and bone resorption ^30^ ^,^ ^31^. 

The TNF-α levels in the negative control group given CMC-Na gel on days 7 and 14 were higher compared to those in the positive control and treatment groups. This was because the CMC-Na gel provided had neutral properties, so it did not provide any effects such as antibacterial, anti-inflammatory, and antioxidant effects. The gel was composed of CMC-Na, propylene glycol, glycerin, and distilled water. These ingredients did not have any therapeutic effect and only functioned to maintain the stability of the gel preparation ^32^. Meanwhile, the treatment group was received cocoa husk extract rich in anti-inflammatory compounds which were proven to reduce TNF-α levels. The inflammatory mediator TNF-α caused connective tissue damage in periodontium and alveolar bone resorption by inducing the production and activation of collagenase enzymes and osteoclasts ^33^. 

The number of osteoclasts showed the same pattern as TNFα levels, where decreases were observed in the number of osteoclasts and TNF-α levels in alveolar bone osteoblasts on day 14 in the negative control group (p < 0.05). *Phorphyromonas gingivalis* produces lipopolysaccharide (LPS) and gingipain which can increase osteoclast activity. Osteoclasts play a role in bone resorption with an excessive expression of RANKL through the activation of T lymphocytes and B lymphocytes in response to the secretion of inflammatory cytokines such as TNF-α and IL-β. This could be due to the decrease in the release of proinflammatory cytokines and the increase in the release of anti-inflammatory cytokines ^34^. 

The treatment group was not significantly different from the positive control group. Therefore, it can be said that both groups had the same effect in terms of inhibiting osteoclastogenesis. Doxycycline contains a group of broad-spectrum antibacterial compounds with potential anabolic effects on bone tissue that work via antibacterial-independent mechanisms. Doxycycline at a low dose plays a role in proliferation, metabolic activity, apoptosis, collagen synthesis, and gene expression relevant to the osteogenic program ^35^.

RANKL expression in the NC group on days 7 and 14 was lower than in the PC and CPH groups. There was a statistically significant decrease in RANKL expression in alveolar bone osteoblasts after the administration of 10% cocoa pod husk extract gel on day 7 and day 14. RANKL is crucial for the complete differentiation of osteoclast precursor cells and periodontal bone resorption. Physiologically it is known that RANKL expression will increase until day 14 and decrease from that point on ^36^ ^,^ ^37^.

The administration of 10% CPH extract gel had the same effect as the administration of 2% doxyxycline gel in inhibiting the osteoclastogenesis process. Cocoa pod husk contains alkaloids, tannins, saponins, polyphenols, flavonoids, tritepernoids, and other compounds, which play an important role in decreasing RANKL expression in alveolar bone osteoblasts in rats with periodontitis. It exhibits antibacterial, antioxidant, and anti-inflammatory activities. The largest bioactive compounds contained in cocoa pod husk extract are polyphenols, including catechin, epicatechin, and isoquercetin ^38^. The effects of catechins can be directly exerted on pre-osteoclasts/osteoclasts or indirectly exerted through the modulation of mesenchymal stem cell (MSC)/pre-osteoclast stromal cell regulation through the activation of the RANKL-OPG system. Catechins can also itensify osteoblastogenesis by enhancing the osteogenic differentiation of MSCs and increasing osteoblastic survival, proliferation, differentiation, and mineralization ^39^.

The present study demonstrated that TNF-α may also mediate osteoclast formation by stimulating RANKL expression in osteoblasts. Receptor activator of nuclear factor-κB ligand plays a role in osteoclast differentiation, function, and survival. The cytokine TNF-α can upregulate RANKL expression in periodontal cells and will increase osteoclast formation, causing periodontal damage. Previous research indicates that TNF closely regulates RANK/RANKL-induced osteoclastogenesis. TNF-α and RANKL promote osteoclastogenesis by upregulating RANK via the NF-κB pathway. TNF acts directly on osteoclast precursors, thus providing a clear target to prevent TNF signaling in states of bone inflammation ^40^. 

Cocoa pod husk extract gel administration at 10% can be an alternative to herbal-based therapy. It significantly decreased the level of TNF-α in gingival crevicular fluid, the number of osteoclasts, and RANKL expression in the alveolar bone osteoblasts of rats with *P. gingivalis*-induced periodontitis on day 7 and day 14. Further studies need to consider evaluating OPG expression, as a coupling with RANKL, to see whether the inhibition of the osteoclastogenesis process is also offset by osteoblast formation. 
